# Comparison between Modelled and Measured Magnetic Field Scans of Different Planar Coil Topologies for Stress Sensor Applications

**DOI:** 10.3390/s18040931

**Published:** 2018-03-21

**Authors:** Robert Gibbs, Gregory Moreton, Turgut Meydan, Paul Williams

**Affiliations:** Cardiff School of Engineering, Cardiff University, Queen’s Buildings, Cardiff CF24 3AA, UK; GibbsRM@cardiff.ac.uk (R.G.); MoretonG@cardiff.ac.uk (G.M.); WilliamsPI1@cardiff.ac.uk (P.W.)

**Keywords:** planar coils, scanning, magnetic imaging, TMR sensor, finite-element modelling

## Abstract

The investigation of planar coils of differing topologies, when combined with a magnetostrictive amorphous ribbon to form a stress-sensitive self-inductor, is an active research area for applications as stress or pressure sensors. Four topologies of planar coil (Circular, Mesh, Meander, and Square) have been constructed using copper track on 30 mm wide PCB substrate. The coils are energized to draw 0.4 A and the resulting magnetic field distribution is observed with a newly developed three-dimensional magnetic field scanner. The system is based on a variably angled Micromagnetics^®^ STJ-020 tunneling magneto-resistance sensor with a spatial resolution of 5–10 µm and sensitivity to fields of less than 10 A/m. These experimental results are compared with the fields computed by ANSYS Maxwell^®^ finite element modelling of the same topologies. Measured field shape and strength correspond well with the results of modelling, including direct observation of corner and edge effects. Three-dimensional analysis of the field shape produced by the square coil, isolating the components **H**_(***x***)_ and **H**_(***z***)_, is compared with the three-dimensional field solutions from modelling. The finite element modelling is validated and the accuracy and utility of the new system for three-dimensional scanning of general stray fields is confirmed.

## 1. Introduction

**Figure 1:**
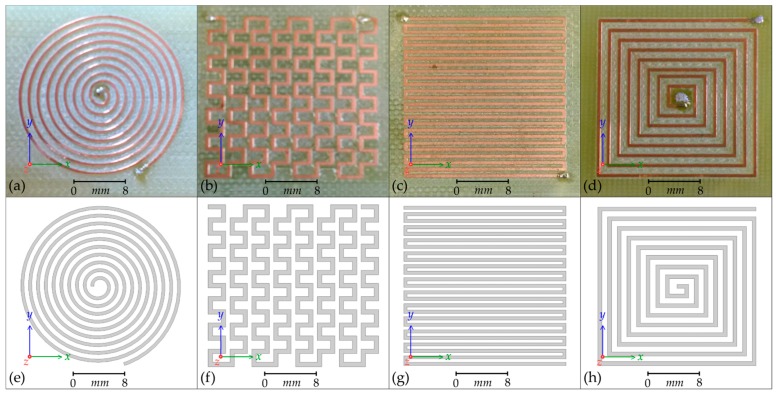
Photographs of the four manufactured planar coils together with the equivalent ANSYS^®^ 3D finite-element models: (**a**,**e**) Circular planar coil; (**b**,**f**) Mesh planar coil; (**c**,**g**) Meander planar coil; (**d**,**h**) Square planar coil. The solder points for the energizing connections can be seen. The wires exit beneath.

Planar coils are generally flat spirals of conductive track mounted on flexible polymer or PCB (printed circuit board) substrate. The shape, number of turns, width, and thickness of track can be varied. Other classes of planar coil are the meander and mesh coils, which do not spiral but alternate direction across the substrate with varying track thickness and displacement (Figure 1). Planar coils have found use in wireless power transfer [1], wireless communication [2], and sensing applications [3], including non-destructive testing [4] and health monitoring [5] where their low profile and robust construction are an advantage [6].

Recent work developing stress sensors utilizing the self-inductive properties of planar coils when combined with magnetostrictive amorphous ribbon has been reported [6,7], where the measured inductance of the system under different stress conditions has been compared with theoretical predictions based on finite-element modelling (FEM) using ANSYS^®^ Maxwell 3D FEM software (ANSYS Inc., Canonsburg, PA, USA). Understanding the physical causes behind the measured induction effects requires a clear knowledge and interpretation of the strength and structure of the magnetic fields produced by each of the topologies. At present, this understanding has been derived solely from FEM. For the first time, corroboration of the shape, strength, and extent of the fields above different planar coil topologies has been made; by the production of two-dimensional field maps (*xy* and *xz*) using both theoretical FEM and practical measurement with a newly developed three-dimensional magnetic field scanner and imaging system. 

**Figure 2:**
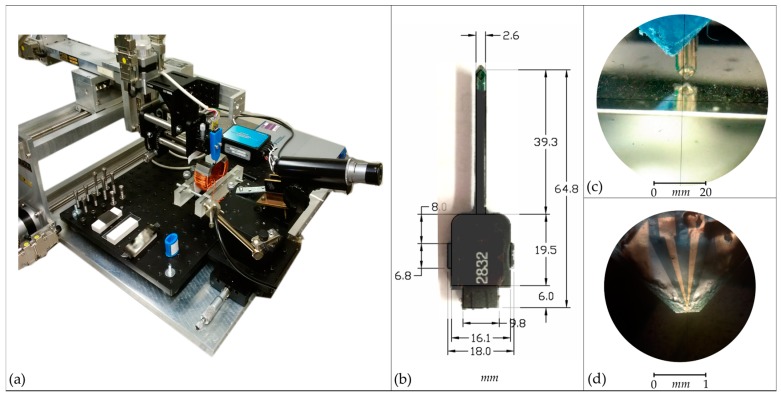
The magnetic field imaging system: (**a**) Built around a three-axis Parker Automation positioning arm (Parker Hannifin Corp, Cleveland, OH, USA), 3D-printed sensor enclosure, precision goniometer, and incorporated microscopic sight; (**b**) The Micromagnetics^®^ STJ-020 tunneling magneto-resistance (TMR) sensor long-probe packaging (Micromagnetics Inc., Fall River, MA, USA); (**c**) The enclosed STJ-020 TMR sensor positioned perpendicular above a sample of (polished) grain-oriented electrical steel (minimum scanning height ***z*_0_** = 12 µm ± 3 µm); (**d**) The Micromagnetics^®^ STJ-020 sensor die with the tip refined to a 7.0 µm ± 0.5 µm tip-edge-to-active-area distance.

The new system (Figure 2) has been developed, as first reported in [8], with the capacity to scan the stray magnetic fields in the thin volume above the surface of a magnetic sample and, through varying the angle of the magnetic sensor, determine the individual vector components of that field.

This work has the dual function of both validating the finite-element modelling of the magnetic fields used to understand the structure of fields above the planar coils, and of confirming the accuracy and utility of the three-dimensional scanning of general stray fields by the new system.

**Figure 3:**
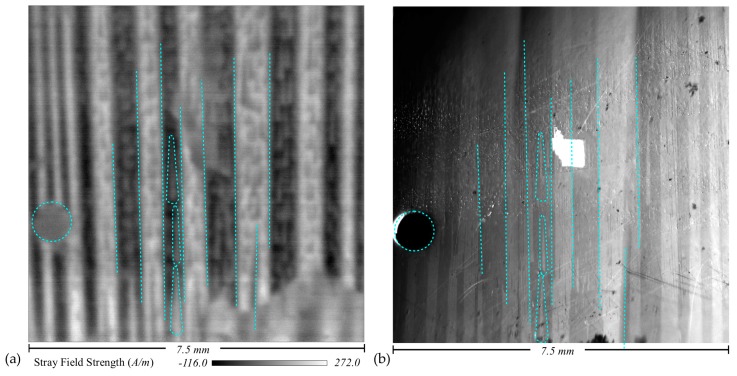
Planar domain observation of stray fields above grain-oriented electrical steel: (**a**) produced by a planar scan with the magnetic field imaging system; (**b**) produced by magneto-optical Kerr effect imaging of the same area of sample. Large bar domains and internal Lancet domains of 50–150 µm are clear. The pinhole used to identify the common area and corresponding magnetic domain features are indicated with blue dotted lines.

Successful planar scans of bar domains and Lancet [9] domains within grain-oriented electrical steel have been validated by magneto-optical Kerr effect (MOKE) imaging of the same area of electrical steel (Figure 3), confirming the system’s spatial accuracy for observing planar domain structures of 50–150 µm. However, the subsequent work [8] on investigating the three-dimensional structure of the stray fields above the sample, whilst promising, was difficult to validate due to the non-uniform structure of the fields being measured. By scanning well-defined and finite-element modelled geometries of magnetic fields, validation of the scanner’s performance can be affirmed which, in turn, validates the measured stray field structure above the non-uniform magnetic samples.

## 2. Materials and Methods 

Four topologies of planar coil have been selected for investigation from [6], which, by convention, are called Circular, Mesh, Meander, and Square coils [10]. The coils have been fabricated by CNC (computer numerically controlled) milling from a 54 mm × 30 mm FR4 PCB with a 35 µm thick copper layer and a track width of 0.5 mm. The dimensions and shape of each coil have been replicated as three-dimensional models within the ANSYS^®^ Maxwell 3D FEM software, given the material properties of copper, and enclosed in an invisible cuboid with the material properties of vacuum; which permits the magnetic fields surrounding the coils to be calculated. Photographs of the manufactured coils and the corresponding ANSYS^®^ models are presented in Figure 1.

The MOKE bench used to validate the scanner’s spatial resolution, previously used for observation of dynamic domain wall movement in amorphous ribbon [11] and grain-oriented 3% electrical steel [12], has a narrow depth of field and produced only a narrow band of in-focus data. The sample needed to be manually stepped through the observing region to collect the data for the entire surface. Figure 3b represents a composite image of these strips, which has been perspective-corrected to account for the ≈60° angle of the MOKE camera and conform it to the planar results produced by the scanner system (Figure 3a).

The three-dimensional magnetic field imaging system (Figure 2) has been developed based on a Micromagnetics^®^ STJ-020 tunneling magneto-resistance (TMR) sensor [13] with a 2 µm × 4 µm active area, which has been refined to be a distance of 7.0 µm ± 0.5 µm from the tip edge (Micromagnetics Inc., Fall River, MA, USA). The sensor is mounted within a custom 3D-printed enclosure and attached to a Parker Automation based three-axis positioning arm [14] with calibrated ±1 µm precision (Parker Hannifin Corp, Cleveland, OH, USA). The sample stage comprises a ThorLabs AMA027/M pitch and roll micrometer adjustable platform [15] to allow the surface to be levelled before scanning (Thorlabs Inc., Newton, NJ, USA). The safe distance of the tip over an uneven sample surface (5 µm ± 2.5 µm) and the refined tip edge give a practical minimum scan height ***z*_0_** = 12 µm ± 3 µm. Similar systems [16] have been used to study geological samples [17] and custom permanent magnetic structures [18], but this is the first time such a system has been developed with a variably angled magnetic sensor and the intention of measuring the weak stray fields from a sample’s intrinsic domain structure. A comprehensive interface has been developed in National Instruments LabVIEW to control the hardware and visualize the resulting data (National Instruments Corporation, Austin, TX, USA).

The four planar coils were mounted on the sample stage and energized to draw 0.40 A ± 0.01 A of direct current using a PowerLine Electronics power supply (PowerLine Electronics, Bergkirchen, Germany). This relatively low current ensured the sensitive STJ-020 sensor was not saturated whilst providing enough field strength to be measured. Scans of a nominal 250 cells × 250 cells were performed with a cell size of 0.1 mm × 0.1 mm at the lowest scanning height sufficient to clear the solder points of the energizing connections. The specific scan heights, ***z*_0_**, and area for each coil are detailed in Table 1. The full scan of the Circular planar coil was necessarily high due to the size of the solder points and thus at a height where differences in the field from each track could not be easily distinguished; consequently, an additional lower scan was made of a region between the two solder points. 

**Figure 4:**
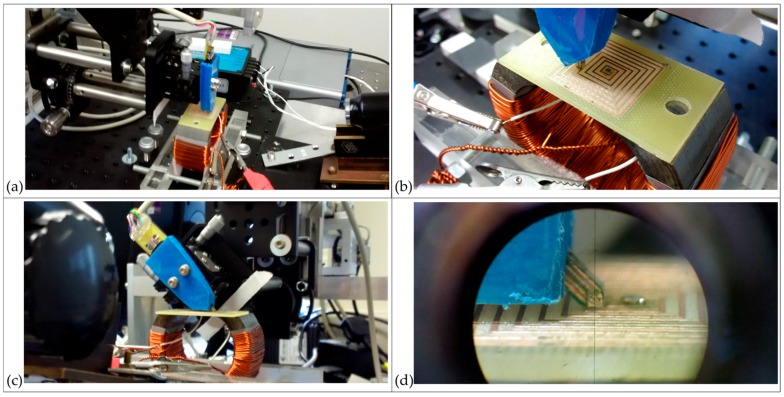
Photographs of the system scanning the square planar coil: (**a**,**b**) with the TMR sensor perpendicular; (**c**,**d**) with the TMR sensor at 45° supported by a precision goniometer. The centre of rotation is precisely aligned to the centre of the sensor’s active area.

The scans of the four coils were performed with the sensor perpendicular to the sample surface. Photographs of the scan being performed above the Square planar coil are presented in Figure 4a,b. The STJ-020 TMR sensor outputs a potential difference proportional to the strength of magnetic field passing through its active area along the axis of the sensor. When perpendicular to the sample surface, this is the magnitude of the z-axis component of the field, **H**_(***z***)_. These voltage data are stored by the system within a three-dimensional matrix which can be output as a .csv file listing the three co-ordinates of the cell centre (in motor steps, 4000 steps per mm) and the voltage in that cell (multiplied by 10^8^ and stored as an integer).

The voltage in each cell comprises the mean of 20 samples taken by the sensor. This number of samples per cell represents an investigated compromise between noise suppression and the amount of time taken to scan. The STJ-020 TMR sensor potential differences (Volts) have been calibrated to field strength (Am^−1^) alongside a Lakeshore 475 DSP Gaussmeter [19] probe within a long solenoid of varied energizing current (Lakeshore Cryotronics Inc., Westerville, OH, USA). The linear calibration factor has been determined as 5.14 mV/Am^−1^ ± 3 µV/Am^−1^. 

Within the ANSYS^®^ finite-element modelling, *xy*-plane slices through the vacuum cuboid were made at distances above the model consistent with the practical scanning heights used for each coil (Table 1). This provided a virtual surface above the modelled coil on which could be projected the magnitude of the components of the calculated fields surrounding the coil, simulating the results of a planar scan at each height. An automatically optimized mesh (maximum length 2 mm) was applied to the cuboid and each slice. This provided a resulting field resolution consistent with the measured data without excessive computing time.

**Figure 5:**
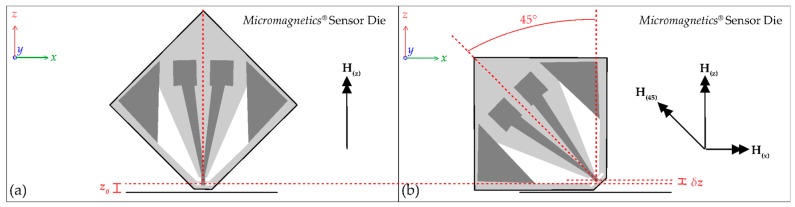
The geometry of the Micromagnetics^®^ STJ-020 TMR sensor die: (**a**) When perpendicular the minimum height of the sensor’s active area ***z*_0_** = 12 µm ± 3 µm; (**b**) At 45° to perpendicular the minimum safe approach of the sensor raises the active area by an initial estimate ***δz*_0_** = 10 µm ± 5 µm. With a rotation of 45° counter-clockwise, the **H**_(**45**)_ component forms from the negative of the **H**_(***x***)_ component and a positive **H**_(***z***)_ component.

To validate the capacity of the system to measure the three-dimensional structure of the magnetic fields by isolating the *z*-axis and *x*-axis components, **H**_(***z***)_ and **H**_(***x***)_, two additional consecutive scans were made of the Square planar coil; another with the sensor perpendicular, and a further corresponding scan with the sensor rotated counter-clockwise at 45° to vertical. Photographs of the angled scan are presented in Figure 4c,d. Only the first half of the coil was scanned to avoid possible collision of the sensor with the central solder point at the lower minimum scan height of ***z*_0_** = 12 µm ± 3 µm. The spatial conformation between the two scans is maintained by the ±1 µm precision of the positioning arm and by the precision goniometer and micrometers used in the system. The axis of rotation was aligned with the centre of the active area of the STJ-020 TMR sensor using the microscope incorporated into the system. A sensor angle of 45° allows for a maximum sampling of **H**_(***x***)_ and also for the lowest active area-to-sample-surface distance (***z*_0_**) possible, due to the shape of the sensor. Explanatory geometry of the sensor is presented in Figure 5. 

There remains a difference in minimum possible sensor-to-surface distance between when the sensor is perpendicular and when it is tilted (***δz*_0_**), which has initially been estimated from microscope measurements to be of the order of 10 µm ± 5 µm.

The ANSYS^®^ Maxwell 3D FEM software allows for simple determination of the magnitudes of **H**_(***z***)_ and **H**_(***x***)_ from the modelling, but to provide comparison of the measured results for the scan with the sensor at 45° the expression

**H**_(**45**)_ = **H**_(***z***)_·cos(45) − **H**_(***x***)_·cos(45) (1)

was used to determine the magnitude of the field which would be expected by a 45° sensor at the height of the modelled scanning plane, named **H**_(**45**)_. Note that the counter-clockwise tilt results in a negative contribution from the *x*-axis component (Figure 5).

From a rearrangement of Equation (1), it can be determined that the expression

**H**_(***x***)_ = **H**_(***z***)_ − (**H_(45)_**/cos(45)) (2)

can be used to calculate **H**_(***x***)_ from the values of **H**_(***z***)_ and **H**_(**45**)_ measured by the scanner system.

To overcome the difficulties in noise levels and spatial and temporal conformity (originally discussed in [8]), each of the **H**_(***z***)_ and **H**_(**45**)_ 250 × 250 matrix data were reduced to a coarser 125 × 125 matrix where the new 0.2 mm × 0.2 mm cells equate to the mean of four of the original 0.1 mm × 0.1 mm cells. This has the advantage of smoothing not only a large amount of spatial non-conformity (albeit with a loss of spatial resolution) but also possible variations in the bias level of the sensor or background transient fields over the time of the scan. Each 0.2 mm × 0.2 mm cell becomes the mean of 80 samples, where the second 40 are taken approximately 5 minutes after the first.

To validate the investigation of the shape of stray fields in the thin volume above a sample’s surface, *xz*-scans were made along a single transect on the Square planar coil. The scan extended 10 mm above the surface of the coil. To maintain consistency with the resolution of the other scans the *x*-axis resolution was 0.1 mm per cell, but to gain detail to the extent of the fields above the surface the *z*-axis resolution was 0.01 mm per cell. The resulting 0.1 mm × 0.01 mm cell is presented as rectangular to maintain the correct aspect ratio between horizontal and vertical distances. To determine the isolated **H**_(***z***)_ and **H**_(***x***)_ components of the field, two consecutive scans were made; one with the sensor perpendicular and one with the sensor at 45° to vertical. Whilst both scans shared the same maximum *z*-axis coordinate, the minimal height (***z*_0_**) of the 45° scan was necessarily 12.5 µm above the minimal height of the perpendicular scan. Thus, allowing for a 2.5 µm error in ***z*_0_** due to surface unevenness, the estimate for ***δz*_0_** can be determined more precisely as 12.5 µm ± 2.5 µm. Using this newly measured offset, reducing the data by averaging four adjacent cells to compensate for some of the noise, and applying Equation (2), the component **H**_(***x***)_ could be determined from the scan.

The expected results calculated from the finite-element modelling of the coil were determined by defining an *xz*-plane slice in the modelled vacuum cuboid at a *y*-axis position matching the transect. The **H**_(***z***)_, **H**_(**45**)_, and **H**_(***x***)_ magnitudes of the calculated fields could again be plotted using Equation (1). Naturally, the finite-element modelling is able to represent the field beneath the coil which is inaccessible to the physical sensor; but, in the case of the thin planar coils, mirrors the field above the sample.

## 3. Results

General information regarding the scan heights, the size of scan, the minimum and maximum measured field strengths of each of the four planar coils, and the greyscale range used were presented in Table 1. The scan heights were governed by the size of the solder points, but were standardized to the least possible of two discrete heights to permit better comparisons to be drawn. The Circular and Square planar coils, as spiral topologies, produce much greater field strengths than the Mesh and Meander. The central loops of spiral topologies receive reinforcing field contributions from each successive concentric outer loop.

**Figure 6:**
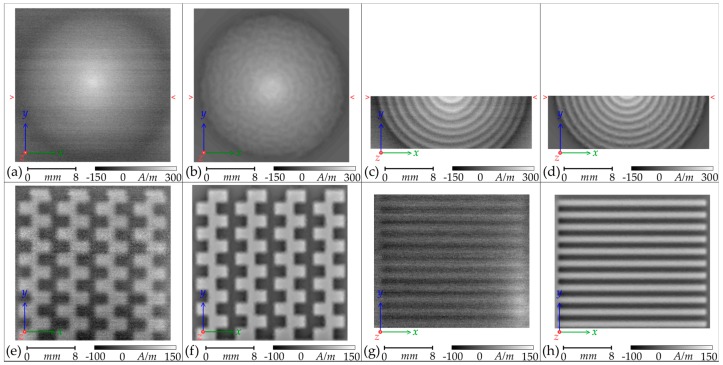
Greyscale maps of the **H**_(***z***)_ amplitudes of three of the manufactured planar coils, energized to draw 0.40 A ± 0.01 A and scanned at the heights presented in Table 1, alongside the equivalent maps calculated by ANSYS^®^ finite-element modelling: (**a**–**d**) Circular planar coil; (**e**,**f**) Mesh planar coil; (**g**,**h**) Meander planar coil. The transect marked by red single-chevrons indicates the path of the two **H**_(***z***)_ amplitude plots presented in Figure 8.

In Figure 6, the results of the scans of the Circular, Mesh, and Meander planar coils are presented alongside the expected results from the finite-element modelling. There is a strong similarity between the measured and the modelled field shape and strength for all three topologies. The scale and position of the greyscale maps presented in Figure 6 have been aligned with the scale and position of the images of the coils presented in Figure 1.

There is significant noise present in the scans, the Micromagnetics^®^ AL-05 power and signal conditioning unit [13] for the STJ-020 sensor is electrically noisy. This limitation has been discussed in [17] and the solution presented was to develop an alternative preamplifier. This is something to be considered in future work but risks damage to the expensive sensor if the bias voltage is not governed correctly. At present, the signal is sufficiently above the noise floor not to be in sufficient detriment as to justify the risk. Reduction of the background systematic interference, indicated by the evident variation (banding) in horizontal scan lines, is a greater priority. Possible sources of the interference include transient background magnetic fields or very slight drifts in the bias voltage applied to the STJ-020 sensor by the AL-05 unit. The bias voltage is applied through a mechanical variable-resistor, which may be prone to slight temperature and vibrational interference.

The granularity within the finite-element modelling maps, particularly Figure 6b, is an artifact of the mesh size. An optimized mesh size with a maximum length of 2 mm obtained this level of detail. A greater maximum mesh size led to a reduction in detail whilst a finer maximum mesh size did not improve the detail, which seems to be a property of modelling these gradual gradients.

At a scan height of 0.5 mm ± 0.01 mm, neither the scan nor the modelling distinguish clearly the individual tracks of the Circular planar coil, leading to a conical overall field amplitude (Figure 6a,b). The closer 0.15 mm ± 0.01 mm scan (Figure 6c,d) illustrates how each concentric ring reinforces sequentially the fields within it. The central peak of the scan is more defined than that of the model and is attributed to the central solder spike of the manufactured coil when compared with the wide flat centre of the finite-element model.

The weaker contrast demonstrated for the Meander and Mesh topologies is, in part, due to the greater (0.25 mm) scan height; however, these topologies produce weaker fields naturally due to the lack of a reinforcing spiral. The checkerboard pattern of the Mesh topology is the result of some field reinforcement within each minor three-sided loop of the mesh. The Meander coil gains no reinforcement; the fields of each leg are counter to each other, resulting in a very weak overall field that is only just discernable above the noise floor. The increase in field strength in the lower right corner of Figure 6g is attributed either to an increase in current and field at the solder point in that corner, or to a difference in copper thickness or relative lifting of the copper track in the manufactured coil.

**Figure 7:**
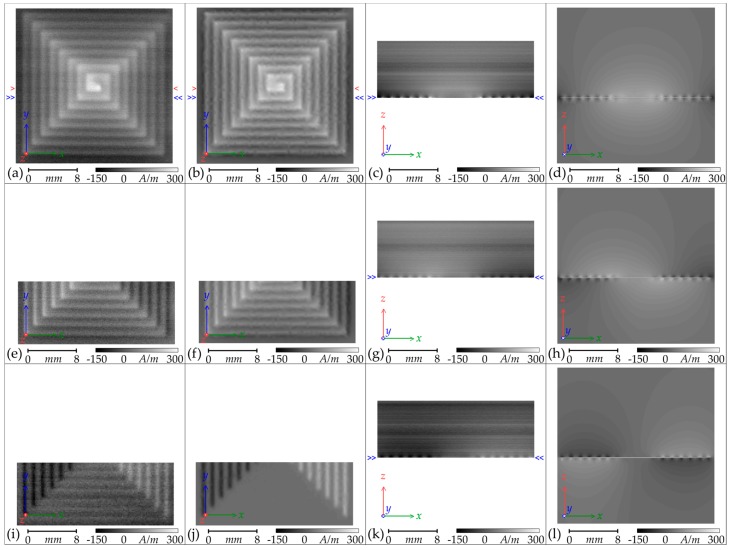
Greyscale maps of the **H** amplitudes of the manufactured Square planar coil, energized to draw 0.40 A ± 0.01 A (Columns 1 and 3), alongside the equivalent maps calculated by ANSYS^®^ finite-element modelling (Columns 2 and 4): (**a**–**d**) **H**_(***z***)_; (**e**–**h**) **H**_(**45**)_; (**i**–**l**) **H**_(***x***)_. Columns 1 and 2 are planar *xy*-maps and Columns 3 and 4 are cross-sectional *xz*-maps along the transect marked by blue double-chevrons. The transect marked by red single-chevrons indicates the path of the **H**_(***z***)_ amplitude plot presented in Figure 9.

The results of scans of the Square planar coil and the analysis to isolate the **H**_(***z***)_ and **H**_(**45**)_ and **H**_(***x***)_ components are presented in Figure 7, alongside the expected results from the finite-element modelling. There is strong similarity between the measured and the modelled field shape for both the planar and the cross-section scans. The scale and position of the greyscale maps have been aligned with the scale and position of the images of the coil presented in Figure 1a,e.

Again, there is significant noise in the scans, particularly in the cross-sectional scans where strong horizontal bands from background interference are present. Nevertheless, the field distributions expected from the finite-element modelling can be seen clearly in the measured cross-sections. The curved “field boundary” lines evident in Column 4 of Figure 7 are an artifact of the discrete grey shading boundaries, but serve to highlight the shape of the field distribution. The modelled fields below the coil are not accessible by practical measurement, but form a mirror of the fields above the coil.

The scans of the **H**_(***z***)_ and **H**_(**45**)_ components have been effectively combined to calculate the **H**_(***x***)_ component, which is presented in Figure 7i,k. There is an increase in granularity due to the reduction in spatial resolution, but the **H**_(***x***)_ component corresponds well with that expected from the ANSYS^®^ modelling. The lower tracks of the Square planar coil are not quite parallel with the scanning path and so some **H**_(***x***)_ signal is present, but a strong **H**_(***x***)_ component away from the centre of the coil on both sides is demonstrated on both the planar *xy*-map and the cross-sectional *xz*-map. Enhancement of field strength at the corners of the squares are clearly demonstrated in both the measured results and the finite-element modelling and this will be discussed further.

**Figure 8:**
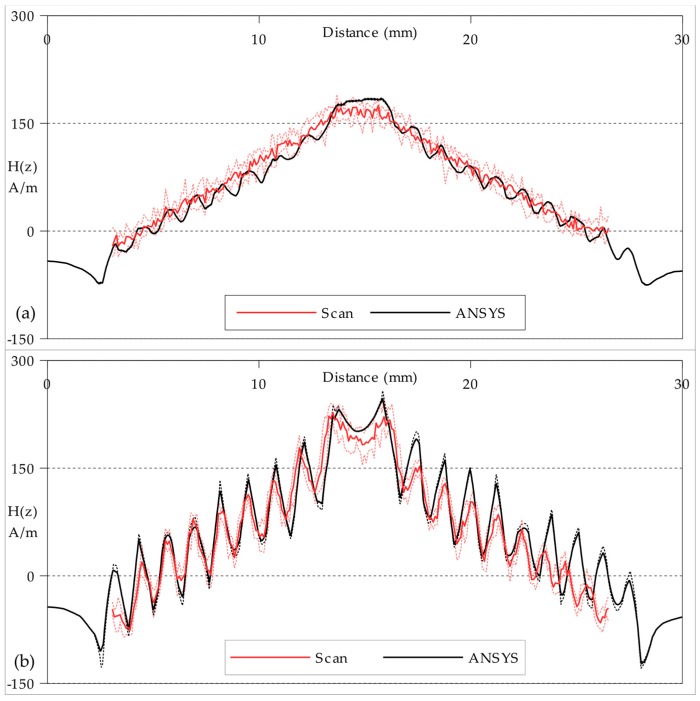
**H**_(***z***)_ amplitude plots of the Circular planar coil along the path indicated by red single-chevrons in Figure 6: (**a**) at a scan height of 0.50 mm ± 0.01 mm; (**b**) at a scan height of 0.15 mm ± 0.01 mm. The ± errors are indicated by dashed lines above and below for both the Scan and ANSYS plots. The distance scale is the distance (mm) along the path from the left edge of the planar coil.

**Figure 9:**
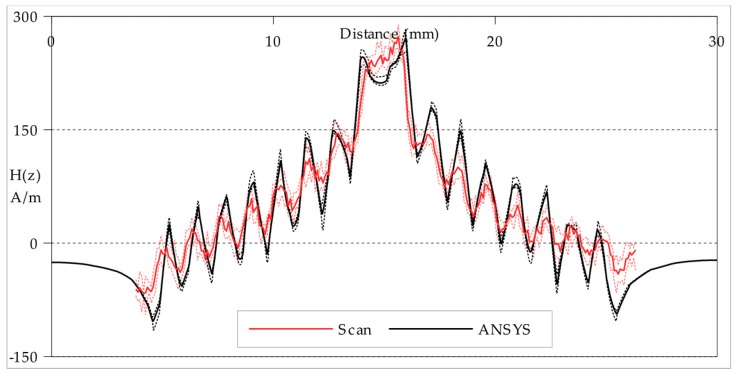
**H**_(***z***)_ amplitude plots of the Square planar coil along the path indicated by red single-chevrons in Figure 7 at a scan height of 0.15 mm ± 0.01 mm. The ± errors are indicated by dashed lines above and below for both the Scan and ANSYS plots. The distance scale is the distance (mm) along the path from the left edge of the planar coil.

The greyscale maps represent well the shape of the **H**_(***z***)_ fields and give an approximate indication of similarity in strength. To better compare the strength of the fields for the Circular planar coil, plots of **H**_(***z***)_ amplitude along the path indicated by red single-chevrons in Figure 6 are presented in Figure 8; and **H**_(***z***)_ amplitude plots of the Square planar coil along the path indicated by red single-chevrons in Figure 7 are presented in Figure 9.

A path with a width of five cells formed the transect between the points marked by the red single-chevrons in Figure 6 and Figure 7. The measured field values plotted in Figure 8 and Figure 9 are the mean of each group of five cells, with the maximum and minimum values plotted as the error range. The finite-element model values are taken directly from the mesh at the *xy*-plane height ***z*_0_** with an error range produced by also calculating the planes at ***z*_0_** ± 0.1 mm. In general, the measured field strengths are slightly lower than expected from modelling. This can be attributed to a combination of the ±0.01 A error in practical current drawn, the ±0.01 mm error in practical scan height and the ±3 µV/Am^−1^ error in the STJ-020 TMR sensor’s calibration; in addition to the statistical error from the background noise level. There is also the possibility of unaccounted power losses within the manufactured planar coils through turn-to-substrate capacitances [20] which are not adequately modelled. There is some spatial discrepancy between the modelled track positions and the milled tracks on the manufactured coils due to the manufacturing tolerances of the CNC milling. The overall field strength is lower the greater the scan height. Though the individual tracks in the Circular coil are not easily distinguished at the greater height (***z*_0_** = 0.5 mm ± 0.01 mm), the plot in Figure 8a indicates that they are still present.

## 4. Discussion

There is a significant increase in field density at the corners of the Square planar coil. These have been expected from the ANSYS^®^ modelling [6,7] but have now been directly confirmed by observation. It has been suggested that these corner effects are caused by “current bunching” around the corner of the square [20]. However, this explanation applies to a high-frequency alternating excitation current (AC) and seems unlikely to apply to direct current (DC) fields which do not induce eddy-currents. Nevertheless, for the direct current used here the effect is still evident in both the finite-element model and the practical scans even though the current might be expected to decrease (the track width increases by a factor of √2 round the corner). An explanation is that the corners of the square behave as the junction of two perpendicular wires such that the fields around each wire combine constructively on both the inside of the corner and, in the opposite direction, the outside.

When used as a self-induction-based sensor system combined with magnetostrictive amorphous ribbon [7], the coils are operated with a high-frequency alternating current where eddy currents are a factor [20]. However, this study now demonstrates that there is also a real and significant DC component to the corner effects, in addition to the AC cause, which could affect the magnetostrictive state of the ribbon and the efficacy of the self-inductor and the sensor.

The magnetic field imaging scanner is currently being developed further to allow for dynamic observation of domain movement in samples with AC external excitation fields. Future work utilizing the planar coils will both confirm the capacity of the new system to scan alternating fields meaningfully and provide further insight into the behaviour of the fields produced by the planar coils during their operation at high-frequency AC as well as the resulting self-induction. The modifications will also allow for the predictable low-frequency AC excitation of the coil, with syncronous detection of the magnetic field, to be used as a method of noise suppression. This current work has been successful in both validating the finite-element modelling of the magnetic fields used to understand the resulting field structure of different planar coil topologies, and of confirming the accuracy and utility of the three-dimensional scanning of general stray fields by the new system.

## Figures and Tables

**Table 1:** The scan height (***z*_0_**) and minimum and maximum measured field strengths, **H**_(***z***)_, above each of the four planar coil topologies when energized to draw 0.40 A ± 0.01 A. The common greyscale ranges ^2^ allow for a better comparison between the resulting magnetic field maps. The mean time taken to scan one cell is 1.414 seconds; the mean scan time for each topology is 24.23 hours.

Planar Coil Topology	Scan Height *z*_0_ (mm)	*x* × *y* (Cells)	Minimum H_(*z*)_ (A/m)	Maximum H_(*z*)_ (A/m)	Map Greyscale Range (A/m)
Circular	0.50 ± 0.01	253 × 250	−85 ± 2	251 ± 2	−150 < H < 300
Circular ^1^	0.15 ± 0.01	253 × 82	−93 ± 2	266 ± 2	−150 < H < 300
Mesh	0.25 ± 0.01	243 × 242	−98 ± 2	140 ± 2	−100 < H < 150 ^2^
Meander	0.25 ± 0.01	260 × 223	−88 ± 2	106 ± 2	−100 < H < 150 ^2^
Square	0.15 ± 0.01	260 × 257	−119 ± 2	295 ± 2	−150 < H < 300

^1^ closer scan between the two solder points; ^2^ narrower range for the weaker fields to enhance contrast.

## References

[1] Low Z.N., Chinga R.A., Tseng R., Lin J. (2009). Design and test of a high-power high-efficiency loosely coupled planar wireless power transfer system. IEEE Trans. Ind. Electron..

[2] Leung S.Y.Y., Lam D.C.C. (2007). Performance of printed polymer-based RFID antenna on curvilinear surface. IEEE Trans. Electron. Packag. Manuf..

[3] Mukhopadhyay S.C. (2004). A novel planar mesh-type microelectromagnetic sensor—Part 1: Model formulation. IEEE Sens. J..

[4] Yamada S., Katou M., Iwahara M., Dawson F.P. (1995). Eddy current testing probe composed of planar coils. IEEE Trans. Magn..

[5] Chen G.-Z., Chan I.-S., Leung L.K.K., Lam D.C.C. (2014). Soft wearable contact lens sensor for continuous intraocular pressure monitoring. Med. Eng. Phys..

[6] Moreton G., Meydan T., Williams P. (2018). Using finite element modelling and experimental methods to investigate planar coil sensor topologies for inductive measurement of displacement. AIP Adv..

[7] Moreton G., Meydan T., Williams P. (2016). A novel magnetostrictive curvature sensor employing flexible, figure-of-eight sensing coils. IEEE Trans. Magn..

[8] Gibbs R., Meydan T., Williams P. Volumetrically scanning the structure of stray-fields above grain-oriented electrical-steel using a variably angled TMR sensor. Proceedings of the 16th IEEE SENSORS Conference.

[9] Shin S., Schäfer R., Charles De Cooman B. (2010). Grain boundary penetration by Lancet domains in Fe-3%Si grain-oriented steel. IEEE Trans. Magn..

[10] Yamada S., Fujiki H., Iwahara M., Mukhopadhyay S.C., Dawson F.P. (1997). Investigation of printed wiring board testing by using planar coil type ECT probe. IEEE Trans. Magn..

[11] Meydan T., Derebasi N., Honda A., Goktepe M. (1992). Dynamic domain motion in amorphous materials under applied tensile stress and controlled magnetisation. J. Magn. Magn. Mater..

[12] Honda A., Goktepe M., Derebasi N., Meydan T., Moses A.J. (1992). Effect of surface roughness and tension on dynamic domain motion in grain oriented 3% silicon iron. J. Magn. Magn. Mater..

[13] Micromagnetics^®^ STJ-020 and AL-05—Product Overview. http://www.micromagnetics.com/products_mtj_f_s.html.

[14] Parker Automation L25i/L50i DIN Rail Stepper Drive—User Guide. http://ph.parker.com/gb/en/products.

[15] ThorLabs Adjustable Platform AMA027/M—Datasheet. https://www.thorlabs.com/thorproduct.cfm?partnumber=AMA027/M.

[16] Shaw G., Kramer R.B.G., Dempsey N.M., Hasselbach K. (2016). A scanning Hall probe microscope for high resolution, large area, variable height magnetic field imaging. AIP Rev. Sci. Instrum..

[17] Lima E.A., Bruno A.C., Carvalho H.R., Weiss B.P. (2014). Scanning magnetic tunnel junction microscope for high-resolution imaging of remanent magnetization fields. IOP Meas. Sci. Technol..

[18] Huber C., Abert C., Bruckner F., Groenefeld M., Muthsam O., Schuschnigg S., Sirak K., Thanhoffer R., Teliban I., Vogler C. (2016). 3D print of polymer bonded rare-earth magnets, and 3D magnetic field scanning with an end-user 3D printer. Appl. Phys. Lett..

[19] Lakeshore Model 475 DSP Gaussmeter—User’s Manual. https://www.lakeshore.com/Documents/475_Manual.pdf.

[20] Kuhn W.B., Ibrahim N.M. (2001). Analysis of current crowding effects in multiturn spiral inductors. IEEE Trans. Microw. Theory Tech..

